# *In vitro* and *in vivo* evaluation of the biofilm-degrading *Pseudomonas* phage Motto, as a candidate for phage therapy

**DOI:** 10.3389/fmicb.2024.1344962

**Published:** 2024-03-15

**Authors:** Prasanth Manohar, Belinda Loh, Dann Turner, Ramasamy Tamizhselvi, Marimuthu Mathankumar, Namasivayam Elangovan, Ramesh Nachimuthu, Sebastian Leptihn

**Affiliations:** ^1^School of Bioscience and Technology, Vellore Institute of Technology (VIT), Vellore, India; ^2^Department of Vaccines and Infection Models, Fraunhofer Institute for Cell Therapy and Immunology (IZI), Leipzig, Germany; ^3^School of Applied Sciences, College of Health, Science and Society, University of the West of England, Bristol, United Kingdom; ^4^Department of Biotechnology, School of Bioscience, Periyar University, Salem, Tamil Nadu, India; ^5^Department of Biochemistry, Health and Medical University, Erfurt, Germany; ^6^Department of Biochemistry and Molecular Biology, University of Southern Denmark, Odense, Denmark

**Keywords:** *Pseudomonas aeruginosa*, phage therapy, bacteriophages, anti-biofilm activity, phage-mammalian cell interaction, *Caenorhabditis elegans*

## Abstract

Infections caused by *Pseudomonas aeruginosa* are becoming increasingly difficult to treat due to the emergence of strains that have acquired multidrug resistance. Therefore, phage therapy has gained attention as an alternative to the treatment of pseudomonal infections. Phages are not only bactericidal but occasionally show activity against biofilm as well. In this study, we describe the *Pseudomonas* phage Motto, a T1-like phage that can clear *P. aeruginosa* infections in an animal model and also exhibits biofilm-degrading properties. The phage has a substantial anti-biofilm activity against strong biofilm-producing isolates (*n* = 10), with at least a twofold reduction within 24 h. To demonstrate the safety of using phage Motto, cytotoxicity studies were conducted with human cell lines (HEK 293 and RAW 264.7 macrophages). Using a previously established *in vivo* model, we demonstrated the efficacy of Motto in *Caenorhabditis elegans,* with a 90% survival rate when treated with the phage at a multiplicity of infection of 10.

## Highlights

- Phages are often evaluated mainly on their ability to kill bacterial hosts.
- One important aspect, however, is often neglected: their ability to degrade biofilms.
- Not all phages are able to disintegrate biofilms.
- While phages can kill planktonic cells, it also has often been observed that phages are not able to infect those that are embedded in biofilms.
- Phage Motto appears to have highly efficient enzymes that degrade biofilms and might, therefore, be a highly valuable therapeutic candidate.

## Introduction

The nosocomial pathogen *Pseudomonas aeruginosa* belongs to the ESKAPE group of bacteria, including *Enterococcus*, *Staphylococcus*, *Klebsiella*, *Acinetobacter*, *Pseudomonas,* and *Enterobacter,* which poses a serious threat to public health. As the rates of antibiotic resistance increase, *P. aeruginosa* infections are becoming more difficult and sometimes impossible to treat, especially due to their tendency to form biofilms in chronic infections. Phage therapy, the use of bacterial viruses to kill bacterial pathogens, may offer a solution (Pirnay et al., 2011; Manohar et al., 2020; Nale and Clokie, 2021; Suh et al., 2022).

Lytic bacteriophages (phages) are bacterial viruses that replicate inside a suitable host and get released through lysis, which destroys the bacterial cell (Ramesh et al., 2021). Phage therapy case studies and clinical trials have been increasing over the last decade as they are often seen as the only suitable alternative to conventional antibiotic therapy, commonly used additionally due to synergetic effects (Gordillo Altamirano and Barr, 2019; Manohar et al., 2022; Dedrick et al., 2023). However, clinical trials have produced mixed results due to the specificity of phages and the rapid development of phage resistance (Jault et al., 2019; Dedrick et al., 2023; Strathdee et al., 2023).

As one of the ESKAPE pathogens, *P. aeruginosa* is a common pathogen known to cause hospital- and community-acquired infections, and it is notorious for being a complication in cystic fibrosis patients (Fujii et al., 2014; Rossi et al., 2021). The increasing rate of antibiotic-resistant *P. aeruginosa* strains is a major concern as they are attributed to high mortality rates in healthcare-associated infections (Lister et al., 2009; Restrepo et al., 2018; Langendonk et al., 2021). Moreover, the ability to form extensive biofilms is a major virulence determinant during pathogenesis, especially in chronic infections (Sibila et al., 2016). Due to their complex composition, biofilms can hinder the efficient diffusion of antibiotics to the target cells, making treatment even more challenging even if the cells are susceptible when not embedded in this polymeric multicomponent matrix. These biofilm-forming pathogenic strains are often found in cystic fibrosis patients but can also cause ventilator-associated pneumonia (Persyn et al., 2019). However, available anti-pseudomonal antibiotics have ceased to be effective in an increasing number of cases as many strains have acquired antibiotic-resistant genes (Botelho et al., 2019). Therefore, an alternative therapy is needed against pseudomonal infections that also demonstrate activity against biofilm-forming cells. This aspect is particularly important because while some phages can effectively kill planktonic cells, biofilms can act as a protective layer to prevent phage infection of embedded bacteria (Erskine et al., 2018; Bond et al., 2021; Behzadi et al., 2022a,b).

In this study, we describe the isolation and characterization of a novel *Pseudomonas* phage, Motto. Motto has a broad host range, infecting more than 50% of the clinical strains we tested, and it can efficiently disrupt biofilms formed by the pathogen. Furthermore, its safe and efficient use was demonstrated using cytotoxicity assays in human cell cultures and in the animal model *Caenorhabditis elegans*.

## Materials and methods

### Bacterial strains

The bacterial strains used in this study were collected from the Hi-Tech Diagnostic Center, Chennai. A total of 50 distinct *P. aeruginosa* isolates collected from clinical samples were used in this study. All the isolates were previously characterized and reported (Manohar et al., 2017, 2021). The isolates were tested for susceptibility to cefotaxime, ciprofloxacin, gentamicin, meropenem, and tetracycline using the micro-broth dilution MIC method following Clinical Laboratory Standard Institute (CLSI) guidelines (CLSI, 2020). The antibiotic sensitivity results were recorded in accordance with the CLSI guidelines.

### Sample collection and isolation of bacteriophages

For the isolation of bacteriophages, sewage water samples were collected from different parts of the Cooum River in Chennai, Tamil Nadu, India (13.0827° N, 80.2707° E). The sewage water samples were collected in containers (up to 1 L), transported to the laboratory, and stored at 4°C. The phage enrichment method was employed to isolate the bacteriophages, following previously described protocols (Manohar et al., 2019). In brief, to the 10 mL of exponentially grown bacterial (host) culture, 30 mL of sewage water sample was added and incubated at 37°C for 20 h. Subsequently, the mixture was centrifuged at 6,000 × *g* for 15 min, and the supernatant was collected. The collected phage lysate was filtered through 0.22-micron syringe filters (≈ 2 mL) and tested for the presence of bacteriophage using the spot test and double agar overlay methods (Manohar et al., 2019).

In brief, in the spot test, a bacterial lawn was prepared using the host bacteria on the Luria-Bertani (LB) agar, and 10 μL of phage filtrate was spotted. The plates were incubated at 37°C for 16 h. The clearance of bacterial growth on the spotted areas denotes the presence of phage activity. For the double agar overlay method, bacteria (200 μL) and phage filtrate (100 μL) were mixed and kept undisturbed for 15 min. Thereafter, soft agar (0.75% w/v, 3 mL) was added, mixed, and poured onto pre-prepared LB agar plates. The plates were incubated at 37°C for 16 h, and the appearance of plaques represented the phage lytic activity. For the screening of bacteriophages, a single plaque was chosen and purified, and the double agar overlay method was repeated thrice.

### Precipitation and purification of bacteriophages

The bacteriophages were precipitated using the polyethylene glycol-sodium chloride (PEG-NaCl) method (Manohar et al., 2019). In brief, 1 M PEG and 0.1 M NaCl were added to the phage filtrate, mixed gently, and incubated at 4°C for 24 h. The precipitant was centrifuged at 15,000 × *g* for 30 min, and 100 μL SM buffer [for 1 L: 5.8 g of NaCl; 50 mL of 1 M Tris–HCl [pH 7.5]; 2 g of MgSO_4_.7H_2_O] was added to the pellet. The phage precipitant was stored at −20°C. To purify the bacteriophages, the sucrose gradient method was used. In brief, varying concentrations of sucrose solution were prepared from 12.5 to 52.5% w/v and layered in descending order of concentration. Subsequently, 1 mL of phage suspension was added at the top and centrifuged at 30,000 × *g* for 1 h, resulting in the concentration of phages as a visible band. The phage band was carefully removed using a micropipette and dialyzed, and the purified phages were stored at −20°C.

### Host range analysis

To study the infectivity of the isolated *Pseudomonas* phage against different bacteria, the selected phage was tested against other 49 clinical strains of *P. aeruginosa* (plus one serving as host), five clinical strains of *E. coli*, three clinical strains of *K. pneumoniae*, three clinical strains of *A. baumannii,* and two clinical strains of *Citrobacter* and *Vibrio*. In brief, the spot test was performed using the phages at 10^3^ PFU/mL against the test bacteria. The lysis of bacteria and the appearance of clear zones represent phage activity. The isolates with the positive spot test results were further chosen for the double agar overlay method. The appearance of plaques indicated phage lytic activity.

### Life cycle studies and burst size

The phage life cycle studies include three different stages as follows: adsorption, latent period, and burst size. To determine the adsorption time, the bacterial cells at 10^6^ CFU/mL were mixed with the phages at the multiplicity of infection of 0.01 (MOI) and incubated at 37°C. Subsequently, 100 μL was withdrawn from the mixture at every 5 min interval for 45 min and diluted in 4.9 mL of LB broth. After incubating for 30 min at 37°C, the non-adsorbed phages were determined using the double agar overlay method. The adsorption curve was plotted based on the number of non-adsorbed phages against time.

A one-step growth experiment was employed to study the latent period, i.e., the time taken for the phages to multiply inside the bacteria, and burst size, i.e., the number of phages released from each infected cell. In brief, the bacterial cells (10^6^ CFU/mL) were mixed with the phages at an MOI of 0.01, and the phages were allowed to adsorb for 30 min at 37°C. Thereafter, the mixture was centrifuged at 12,000 × *g* for 5 min, 10 mL of LB broth was added to the pellet, and it was incubated at 37°C. Subsequently, at every 10 min interval, the samples were taken and titrated against the host bacterium. Both the latency period and burst size were plotted against time (in min). All the data are presented as mean ± standard deviation (SD) of at least three independent experiments.

### Stability studies

The stability of the phages was determined at different pH (Pirnay et al., 2011; Fujii et al., 2014; Gordillo Altamirano and Barr, 2019; Jault et al., 2019; Manohar et al., 2020; Nale and Clokie, 2021; Ramesh et al., 2021; Manohar et al., 2022; Suh et al., 2022; Dedrick et al., 2023; Strathdee et al., 2023) and temperature (20–70°C) values, following previously described protocols (Feng et al., 2021). In brief, the phage suspension adjusted to a titer of 10^6^ PFU/mL was incubated under various physical conditions. The reduction in phage titer was analyzed after 60 min using the overlay plaque assay. In brief, the pH stability studies were performed using SM buffer, and the phage lysates were incubated at varying pHs ranging from 1 to 12. For thermal stability studies, the phage lysates were incubated at 20 to 70°C for 60 min and tested for phage activity. All the data are presented as mean ± standard deviation (SD) of at least two independent experiments.

### Transmission electron microscopic analysis

To morphologically characterize the bacteriophage structure, the phages were negatively stained using uranyl acetate and visualized under TEM (FEI-TECNAI G2-20 TWIN, Bionand, Spain) at the VIT-TEM facility (Manohar et al., 2019). In brief, in a copper grid, 2 μL of purified phages at 10^8^ PFU/mL was added and allowed to adsorb for 10 min. The excess samples were removed, and the grid was dried. Staining was performed by adding 2% (w/v) uranyl acetate. The copper grid was washed three times with distilled water to remove excess stain and allowed to dry for 30 min prior to examination.

### Genome sequencing and analysis

Initially, the phage DNA was isolated using the phenol-chloroform method (24:1) and precipitated using ethanol (Manohar et al., 2018). The phage genome was sequenced using the Illumina HiSeq platform. The complete genome annotation was published elsewhere and can be read here (Manohar et al., 2022).

### Comparative genomics and phylogenetics

All phages classified as species within the family *Drexlerviridae* in the ICTV Metadata Resource version 37^1^ were retrieved from GenBank. Nucleotide sequence similarity between phages was compared using VIRIDIC with default settings. To determine appropriate core genes conserved within the family for phylogenetic analysis, phage genomes were first reannotated using Prokka version 1.14.5 (Seemann, 2014) with a custom hmm pVOGs database to provide consistent gene calls. The pan-genome analysis was conducted with the predicted protein-coding sequences using PIRATE (Bayliss et al., 2019) with parameters of 35, 45, and 55% identity. The maximum likelihood phylogenetic trees were constructed from ClustalO (Sievers and Higgins, 2018) alignments with IQTree2 (Minh et al., 2020). IQTree2 was run with ModelFinder (Kalyaanamoorthy et al., 2017) to select the most appropriate evolutionary model according to the Bayesian information criterion with 1,000 Ultra-fast bootstrap replicates (Hoang et al., 2018) and the SH-aLRT test. The trees were rooted using an out-group (*Dhillonvirus* JG1) before being annotated with ITOL (Letunic and Bork, 2021).

### Biofilm clearance assay

To study biofilm formation and the anti-biofilm activity of phages, a microtiter plate assay was performed. In brief, the *P. aeruginosa* strains (*n* = 32, based on phage activity) were grown in LB broth in polystyrene microtiter plates for 24 h, at 37°C without shaking. The samples were then fixed with 100% methanol, stained with 1% crystal violet (CV), and washed twice with PBS, before reading the OD at 595 nm (BioTek, India). Biofilm formation was recorded as weak, moderate, and strong based on the OD values in comparison with the control (range: weak with OD of <1.0, moderate with OD of 1.0–2.0, and strong with OD of >2.0).

To study the anti-biofilm activity of phages, 10 strong biofilm-forming (based on OD) *P. aeruginosa* isolates were chosen (*P. aeruginosa* strains 01, 08, 11, 16, 27, 32, 35, 37, 42, and 47). The anti-biofilm activity of the phages was determined after adding the phages (10^6^ PFU/mL) to the biofilm, which was allowed to form over a period of 24 h. Following a 24 h co-incubation of phages with bacteria at 37°C, the CV-staining protocol was performed, and the OD values were determined. All the data are presented as mean ± standard deviation (SD) of at least three independent experiments with two data points each.

### Cytotoxicity of bacteriophage on mammalian cells

#### Maintenance of mammalian cell lines

The human embryonic kidney (HEK 293) cell lines were obtained from the VIT Cell Culture facilities, SBST (George et al., 2019). The cell lines were grown in Dulbecco’s Modified Eagle’s Medium (DMEM) containing 10% (v/v) fetal bovine serum (FBS) and 5 mL/L (100x) antibiotic solution, which contains 100 U/mL penicillin, 100 mg/mL streptomycin, and 25 μg/mL Amphotericin B. The cells were maintained at 37°C in 5% atmospheric CO_2_ and 95% air. The cells were grown to 80–90% confluence in a T25 flask. RAW 264.7 macrophage cells, along with another cell line (established murine macrophage cell lines, ATCC), were cultured in complete DMEM with high glucose and supplemented with 10% (v/v) FBS, 100 U/mL penicillin, and 100 mg/mL streptomycin, and pH was adjusted to 7.4 The cells were maintained at 37°C in 5% CO_2_ until they reached 70 to 80% confluence. The cells (3 × 10^5^ cells/mL) were then cultured overnight for further assays.

#### Testing the bacteriophage-mammalian cell line interaction

The purified bacteriophages at 10^6^ PFU/mL were used to treat the mammalian cell lines, HEK 293 and RAW 264.7 macrophages, to determine the cytotoxicity (Porayath et al., 2018). In brief, 5×10^5^ cells were seeded into wells of a six-well plate and maintained at 37°C, 5% in DMEM with 10% FBS. After 24 h, the cells were washed with PBS and treated with phage suspension without FBS. HEK293 cells and RAW 264.7 macrophages treated with Triton X-100 served as a positive control. To count the cells using a hemocytometer, 100 μL of cell suspension was taken, and 100 μL of 0.4% trypan blue was added. A 20 μL of cell suspension was filled under the cover glass, and the cells were counted under 10x magnification.

### Testing the efficacy of phage in the animal infection model *Caenorhabditis elegans*

#### *Caenorhabditis elegans* maintenance

Bristol N2 (wild-type) *C. elegans* was used in this study, and the nematode was maintained and propagated on nematode growth media (NGM; 17 g agar, 3 g NaCl, 2.5 g peptone, 0.5 mL of 1 M CaCl_2_, 1 mL of 5 mg/mL cholesterol, 1 mL of 1 M MgSO_4_, and 25 mL KH_2_PO_4_ buffer [pH 6.0] per liter) plates, which carried *E. coli* OP50 as a source of food at 20°C by standard protocols (Brenner, 1974). Age-synchronized L4 worms were exposed to TSB, *P. aeruginosa,* and *Pseudomonas* phage in a 96-well microtiter plate containing M9 buffer in each of the wells for the phage efficacy assay.

#### Phage efficacy assay

A liquid-based assay to study the efficacy of phages against pathogenic bacteria was previously developed by our research team (Manohar et al., 2022). To test the efficacy of *Pseudomonas* bacteriophage in recovering the nematodes from pseudomonal infection, two types of studies were performed, i.e., therapeutic treatment and prophylactic treatment. For a liquid-based assay, a 96-well microtiter plate was filled with M9 buffer to which overnight culture of *E. coli* OP50 or an equivalent amount of *P. aeruginosa*, with or without phage, was added. Subsequently, 20 mature L4 nematodes were transferred into the solution (Manohar et al., 2022). The total volume in the microtiter plate was maintained at 100 μL. To test the phage efficacy, different concentrations of *P. aeruginosa* were used to test the infectivity. Accordingly, overnight cultures of bacteria were diluted to 10^5^ CFU/mL (based on our previous study), and the efficacy of phage treatment was studied using various concentrations of phages, i.e., 10^5^, 10^6^, and 10^7^ CFU/mL. Therefore, the study included bacteria and phage concentrations at the ratios of 1:1 (10^5^ CFU/mL: 10^5^ PFU/mL), 1:10 (10^5^ CFU/mL: 10^6^ PFU/mL), and 1:100 (10^5^ CFU/mL: 10^7^ PFU/mL).

Group 1 (control) consisted of M9 buffer (60%) with *E. coli* OP50 at 10^5^ CFU/mL (40%) and 20 nematodes. Group 2 consisted of M9 buffer, 20 nematodes, and no bacteria. Group 3 consisted of M9 buffer (60%), TSB (40%), and 20 nematodes. Groups 2 and 3 were experimental controls. Group 4 (infection control) consisted of M9 buffer (60%), *P. aeruginosa* (40%), and 20 nematodes. Group 5 (heat-inactivated bacteria) consisted of M9 buffer (60%), heat-inactivated *P. aeruginosa* (40%), and 20 nematodes. Group 6 (phage toxicity test) consisted of M9 buffer (60%), *Pseudomonas* phage Motto (40%), and 20 nematodes. Group 7 (therapeutic treatment group) consisted of M9 buffer (40%), *P. aeruginosa* (30%), and 20 nematodes; *Pseudomonas* phage Motto (30%) was added after 2 h of exposure to bacteria. Group 8 (prophylactic treatment group) consisted of M9 buffer (40%), *Pseudomonas* phage Motto (30%), and 20 nematodes; *P. aeruginosa* (30%) were added after 1 h. The plates were incubated at 20°C, and the survival of the nematodes was monitored every 24 h for 5 days. The results were evaluated based on live nematodes (moving) and dead nematodes (lack of movement). All the experiments were repeated three times for statistical significance.

To analyze the presence of phages inside the nematodes or uptake of phages by the nematodes (Manohar et al., 2022), the phage numbers were determined as follows: In brief, after 4 days, 10 nematodes were removed from groups 5, 6, and 7, corresponding to phage only, therapeutic treatment, and prophylactic treatment, respectively, to determine the phage titer using the double agar overlay method. In brief, the nematodes were washed thrice with buffer, vortexed, ground (with mortar and pestle), and centrifuged at 10,000 x *g* for 5 min. The supernatant was used to determine the phage titer (Manohar et al., 2022).

### Statistical analysis

All the experiments were performed at least three times for statistical significance, and the data were presented as the mean ± standard deviation (SD). For nematode studies, survival curves were plotted using the Kaplan–Meier method, and the log-rank test was used to calculate the difference in survival rates using GraphPad Prism software 9.0 (GraphPad Software, Inc., La Jolla, United States). *p < 0.05* was considered statistically significant using the log-rank test.

## Results

### Isolation of *Pseudomonas* infecting phages

**Figure 1:**
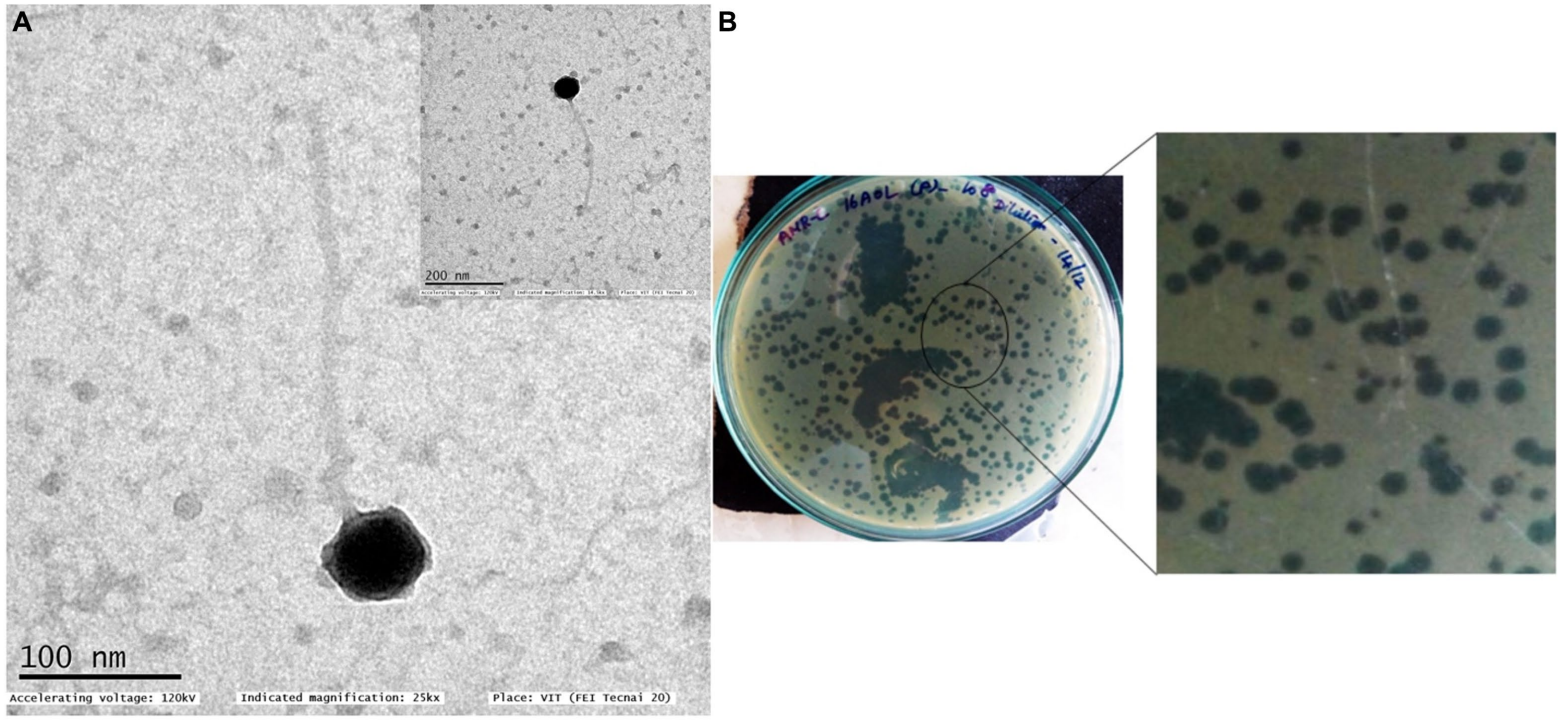
Transmission electron micrograph of *Pseudomonas* phage Motto **(A)** and morphology of plaques formed against *P. aeruginosa* isolate PA01 **(B)**. Two different plaque morphologies originated from the same phages. TEM morphology shows that the phage exhibits a T1-like or siphovirus morphology with an icosahedral head and long non-contractile tail.

For this study, we first screened for phages using 50 clinical *P. aeruginosa* isolates with a mucoid phenotype as “bait”; most of the isolates were found to be multidrug-resistant (Supplementary Table S1). From five different environmental water samples, three phages infecting three different hosts were isolated and chosen for further studies. When analyzing the host range of the phages, we found that it had the ability to infect the majority of tested clinical *P. aeruginosa* strains 32/50 (64%) (Supplementary Figure S2). This phage was named Motto and was isolated from Cooum River water in Chennai, India. The plaque morphology was identical in all strains, forming clear plaques without any halo of 1.5–2 mm after overnight incubation (Figure 1). Although there were some differences in plaque morphology after overnight incubation, the plaques were originated from a single phage (Figure 1B). In our case, the exposure to phage Motto resulted in complete lysis of host bacteria on solid and liquid media for all phage-sensitive isolates (*n* = 32); on the plates, no growth was observed, and in liquid, visibly clear cultures were seen, even after extended incubation times. Under the conditions we tested, we did not observe phage-resistant phenotypes both in agar overlay plates and in growth kinetics (Supplementary Figure S3). Motto was found to be host specific because no plaques or lysis was observed against *E. coli, K. pneumoniae*, *A. baumannii, Citrobacter,* and *Vibrio* sp.

### *Pseudomonas* phage Motto morphology is T1-like

Negative staining and TEM analysis allow the morphological characterization of phages. Using this technique, we found that Motto possesses a T1-like or siphovirus morphology, with a long, flexible tail morphologically similar to the members of the family *Drexlerviridae*. The icosahedral head is symmetrical and approximately 57 ± 1 nm in size, while the long non-contractile tail is 255 ± 1.5 nm in length (Figure 1).

### Replication and adsorption of phage Motto are rapid processes

**Figure 2:**
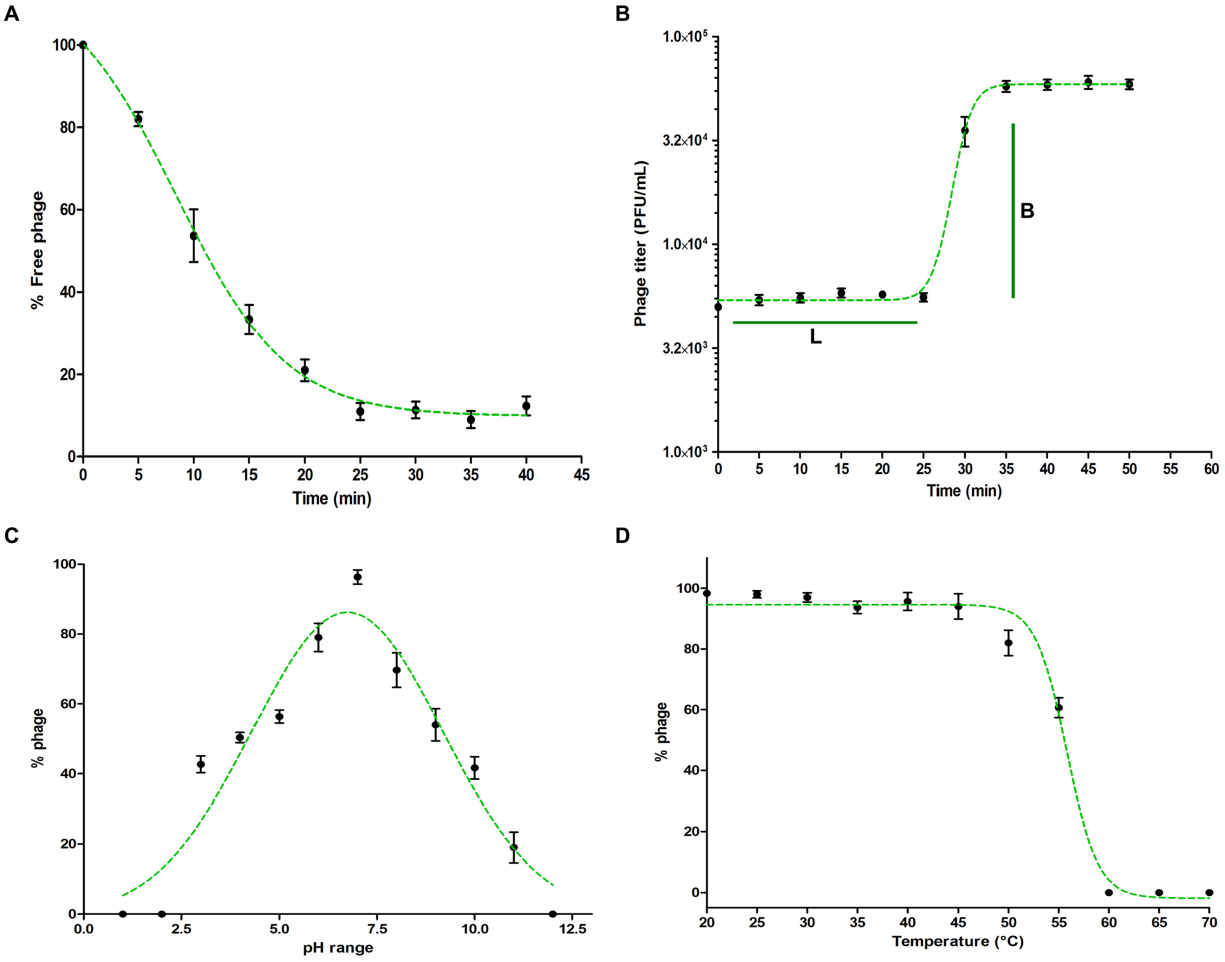
Characterization of *Pseudomonas* phage Motto. **(A)** Adsorption of phage to its host *P. aeruginosa*. The graph represents the % reduction in phage titer against the time (in min). The adsorption plots were fitted using an exponential decay reaction. **(B)** One-step growth curves of *Pseudomonas* phage Motto. The graph represents the change in titer against the time (in min). The plots were fitted using the sigmoidal fit. **(C)** pH stability of *Pseudomonas* phage Motto; the graph represents the % stability at varying pH conditions. The optimal pH stability was determined by plotting the Gaussian fitting function. **(D)** Thermal stability of *Pseudomonas* phage Motto; the graph represents the stability at different temperatures (°C). The stability plots were fitted using the sigmoidal fit. All the experiments were performed at least three times for statistical significance, and the data are presented as the mean ± standard deviation (SD).

The adsorption kinetics of *Pseudomonas* phage Motto to the host surface was rapid, with approximately 50% of the phages attaching to the host surface within approximately 10 min and 90% of phages being adsorbed to host cells within approximately 25 min (Figure 2A). After adsorption, phage and host machinery facilitate the translocation of the viral genome followed by the replication of the virus. In the case of phage Motto, this process requires approximately 30 min at 37°C (Figure 2B). A complete lysis of the culture, producing a transparent lysate, was observed at approximately 40 min. The burst size was calculated with 80 phage particles per host cell on average.

### Motto is stable under non-physiological conditions

Therapeutic phages have to be stable for storage and application. Therefore, we studied Motto’s stability at elevated temperatures and non-neutral pH values. We found that phage Motto remains active within pH ranges of 3 to 11 with optimum stability observed at a pH value of 6.7 (Figure 2C). The thermal stability showed that Motto remains stable up to 55°C when incubated for 1 h, whereas it is completely inactivated at 60°C (Figure 2D).

### Genomic characterization of phage Motto

**Figure 3:**
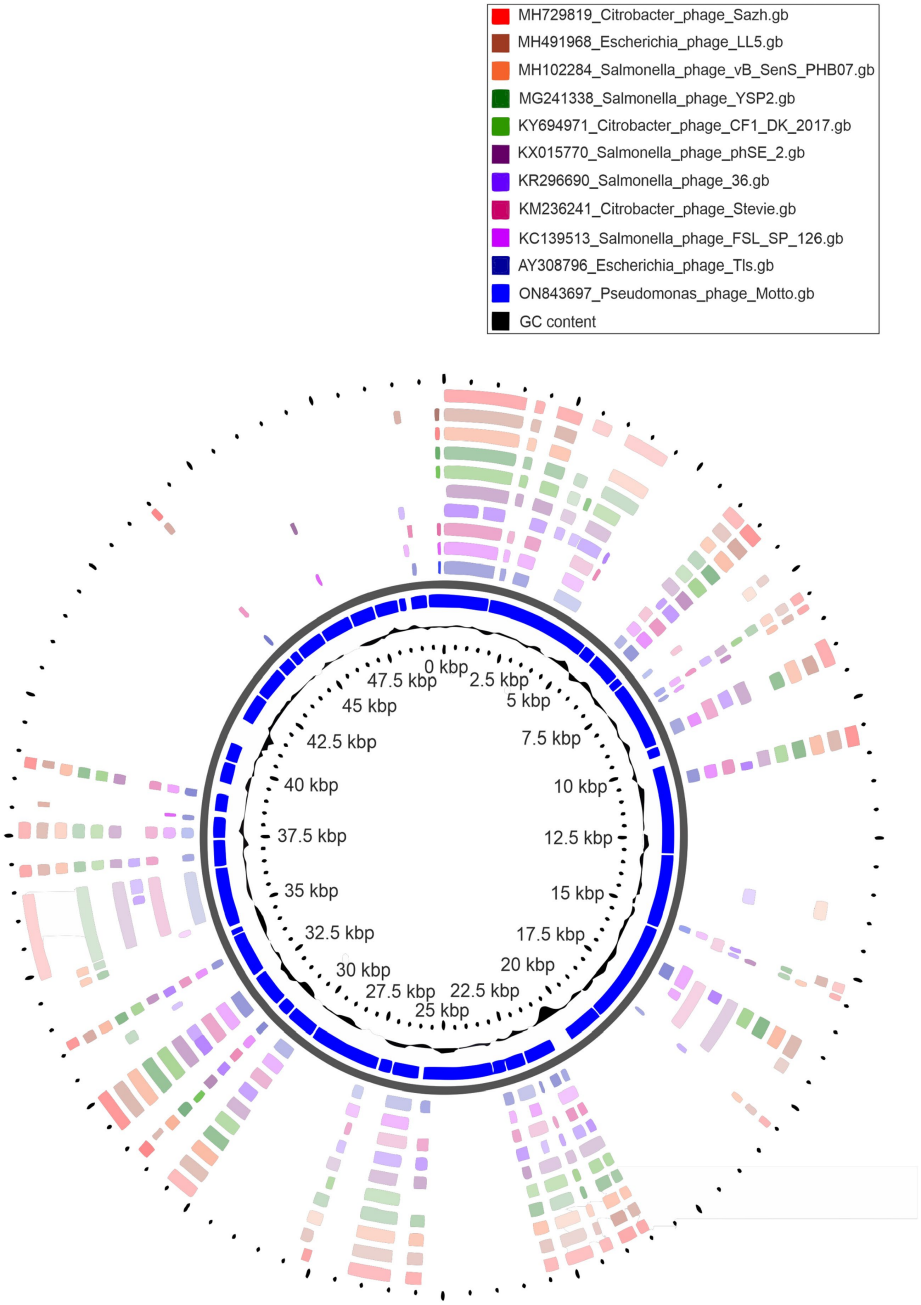
Circular genome representation of *Pseudomonas* phage Motto with the 10 closest related phages using CGViewer.

The Motto genome is a linear double-stranded DNA molecule with a size of 49,960 base pairs and a G + C content of 45% (NCBI accession number is ON843697) (Figure 3). A complete genome announcement is published elsewhere (Manohar et al., 2022). Supplementary Table S2 shows the genes coding for proteins with putative functions, separated by their respective roles.

### Taxonomic classification of *Pseudomonas* phage Motto

Initial analysis with BLASTn identified similarities to members of the *Tlsvirus*, family *Drexlerviridae*. To assess this relationship, nucleotide sequences of phages classified within the family were subjected to pairwise comparison using VIRIDIC (Supplementary Figure 4). The analysis of the nucleotide similarity between Motto and 167 species assigned to this viral family recapitulates the assigned genera and indicates that Motto represents a new species and genus according to current ICTV demarcation criteria (Turner et al., 2021).

The pan-genome analysis revealed that a total of 10 gene products are conserved in all members of the *Drexlerviridae*. This number is likely to be greater when taking into account that the sequences are not colinear and that some genes were not called due to being split across the genome ends. Of the 12,363 proteins, 12,166 formed a total of 311 groups consisting of two or more representative sequences and 197 were unique singletons. Motto shares a maximum of 75% of proteins with *Klebsiella* phage GML-KpCol1 (MG552615) and a minimum of 44% with other *Drexlerviridae* species.

**Figure 4:**
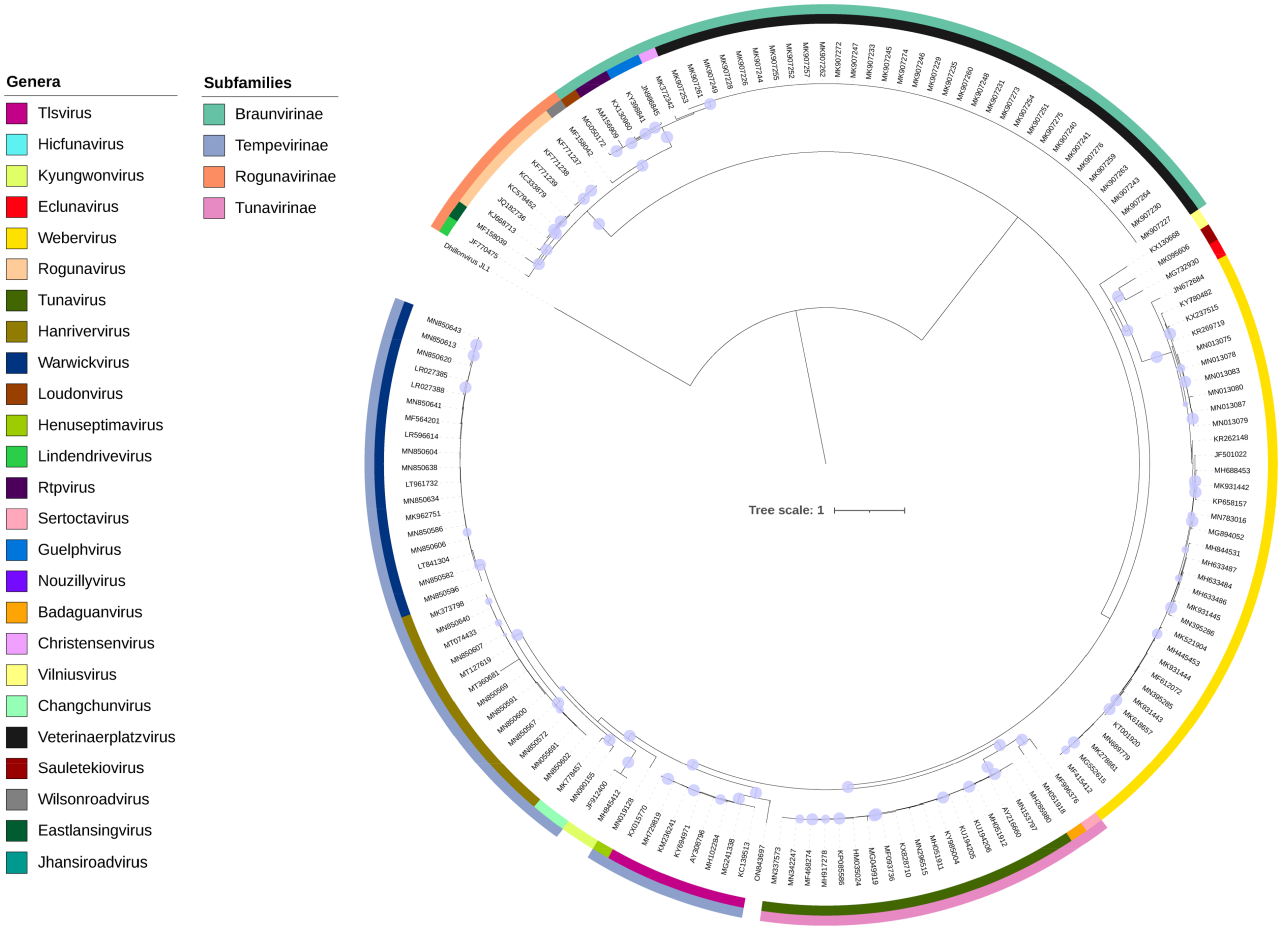
Maximum likelihood phylogenetic tree of proteins conserved across *Drexlerviridae*. Models were predicted for each alignment (TerS, Q.yeast+I + G4; TerL, LG + G4; major capsid protein, WAG+R3; tail assembly, LG + G4; tail completion protein, WAG+R4; tail tip protein Q.pfam+F + R5; major tail protein, WAG+G4; tail terminator protein, WAG+G4; helicase, WAG+G4 and tape measure protein, Q.pfam+F + R4). Ultrafast bootstrap support (1,000 replicates) > =95% is shown as filled circles. Colors on the inner and outer rings denote genera and subfamilies according to the key.

For the phylogenetic analysis (Figure 4), a partitioned maximum-likelihood tree was constructed using 10 gene products conserved in all *Drexlerviridae* members: Terminase small subunit, terminase large subunit, major capsid protein, tail assembly protein, tail completion protein, tail tip protein, major tail protein, tail terminator protein, helicase and tape measure protein. The topology of the tree revealed clades that were largely congruent with defined genera and subfamilies. Some exceptions were noted; first, the genus *Kyungwonvirus,* comprising *Cronobacter* phages Esp2949-1 (JF912400) and CS01 (MH845412), separates the *Tempevirinae.* Second, the tree topology does not appear to support the inclusion of *Shigella* phage Sd1 (MF158042, *Wilsonroadvirus*) within the *Rogunavirinae* subfamily. The taxonomy of this family will require reassessment in the near future, to include new isolates deposited in the INSDC since its inception in 2019 (ICTV proposal 2019.100B; https://ictv.global/ictv/proposals/2019.100B.zip) and to allow a more detailed examination of the minor discrepancies observed here. Collectively, based on nucleotide similarity, a number of shared proteins, and phylogenetic analysis, we propose that Motto represents a new species and genus within *Drexlerviridae*.

### Phage-mediated reduction of biofilms

**Figure 5:**
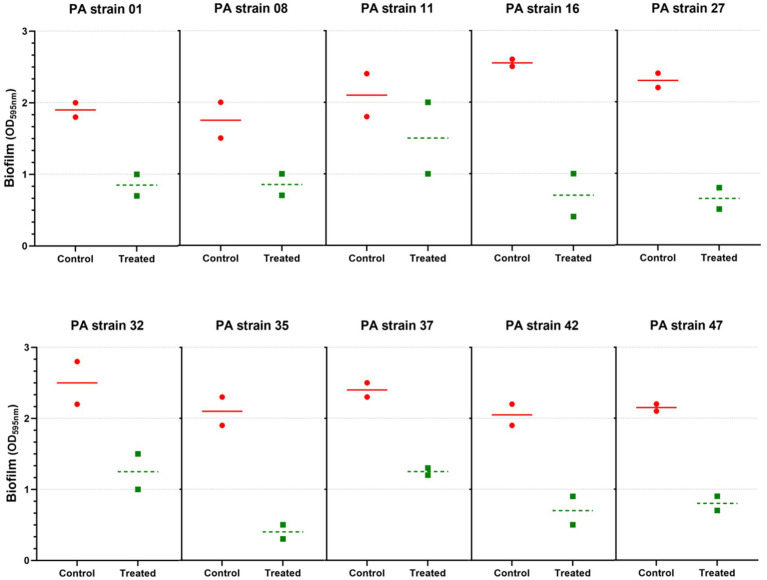
Anti-biofilm activity of *Pseudomonas* phage Motto against different strains of *P. aeruginosa*. A total of 10 strong biofilm producers were chosen and treated with phage (10^6^ PFU/mL) for 24 h. The results were compared between the untreated and treated groups. All the experiments were performed at least three times for statistical significance, and the data are presented as the mean ± standard deviation (SD).

The *in vitro* biofilm assay using the microtiter plate method showed that 10 out of 32 isolates were strong biofilm producers (*P. aeruginosa* strains 01, 08, 11, 16, 27, 32, 35, 37, 42, and 47). Of the 10 *P. aeruginosa* isolates tested, biofilm formation was substantially reduced in all, and at least 75% reduction was observed (Figure 5). Importantly, our study showed the reduction of strong biofilms after 24 h of treatment, under the conditions tested.

### Motto does not exhibit cytotoxic effects when applied to mammalian cells

**Figure 6:**
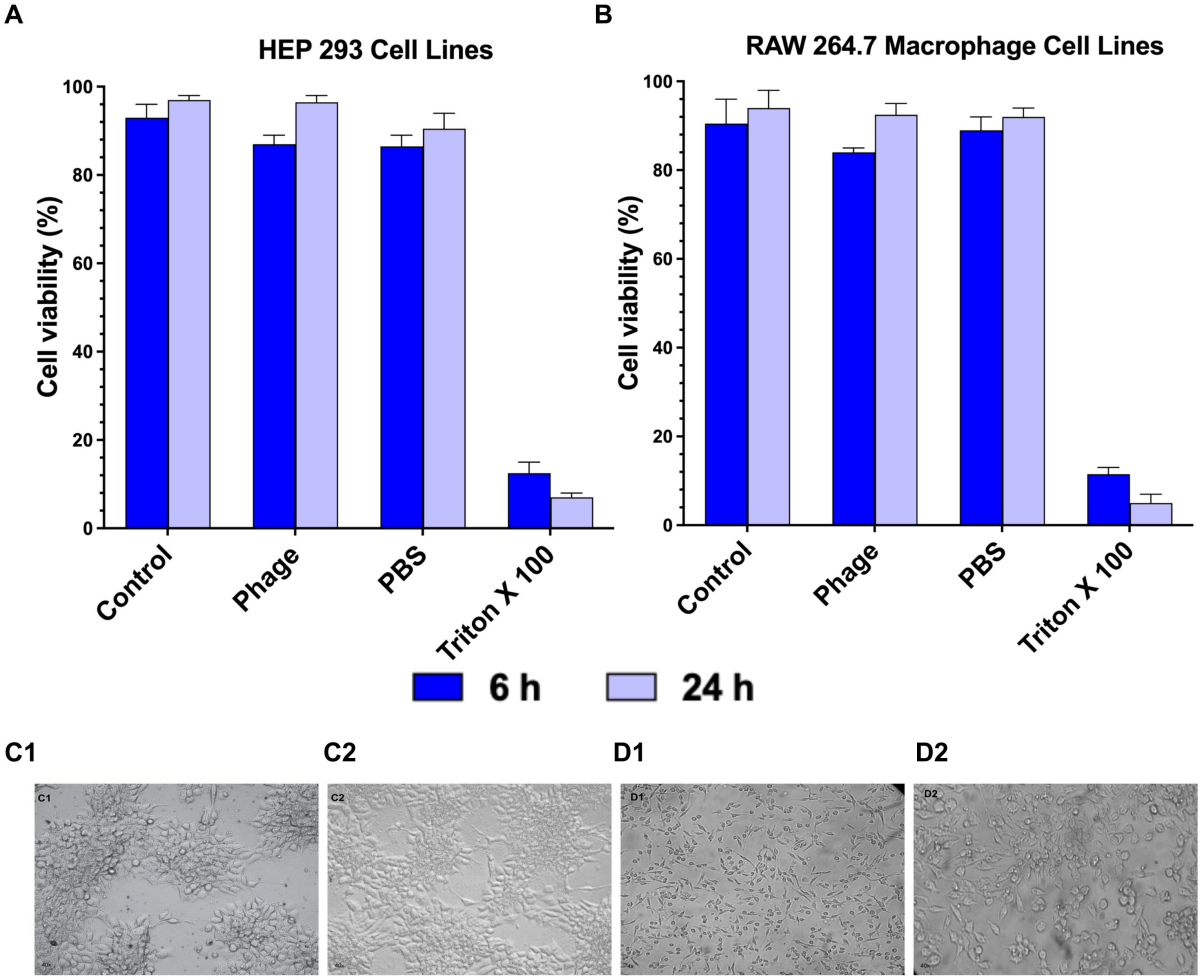
Cytotoxicity of *Pseudomonas* phage Motto on mammalian cells. The cytotoxicity was studied using **(A)** HEP 293 cell lines and **(B)** Raw 264.7 Macrophage cell lines (5 × 10^5^ cells/well) using cell viability studies. The graphical representation of the viability of mammalian cells with and without phage treatment shows that the phage treatment did not affect the viability in comparison with the positive control (1% Triton X-100). (C—HEK 293 cell images, C1—Control, C2—Phage treated, D—RAW 264.7 cell images, D1—Control, and D2—Phage treated). Cell images show that there is no change in morphology after phage treatment (C2 and D2) as compared to the controls. All the experiments were performed at least three times for statistical significance, and the data are presented as the mean ± standard deviation (SD).

*Pseudomonas* phage Motto was tested for toxicity with the mammalian cell lines, HEK 293 and RAW 264.7 macrophages, when exposed to the virus particles for 24 h. When the cells were exposed to a solution containing phages, no cytotoxic effect was observed using cell viability counts (Figures 6A,B). Phage-treated cells and normal cells exhibited no difference in cell morphology (Figures 6C,D) after 6 and 24 h of incubation in our experiments and under the conditions we tested.

### Application of Motto to nematodes results in reduced mortality

**Figure 7:**
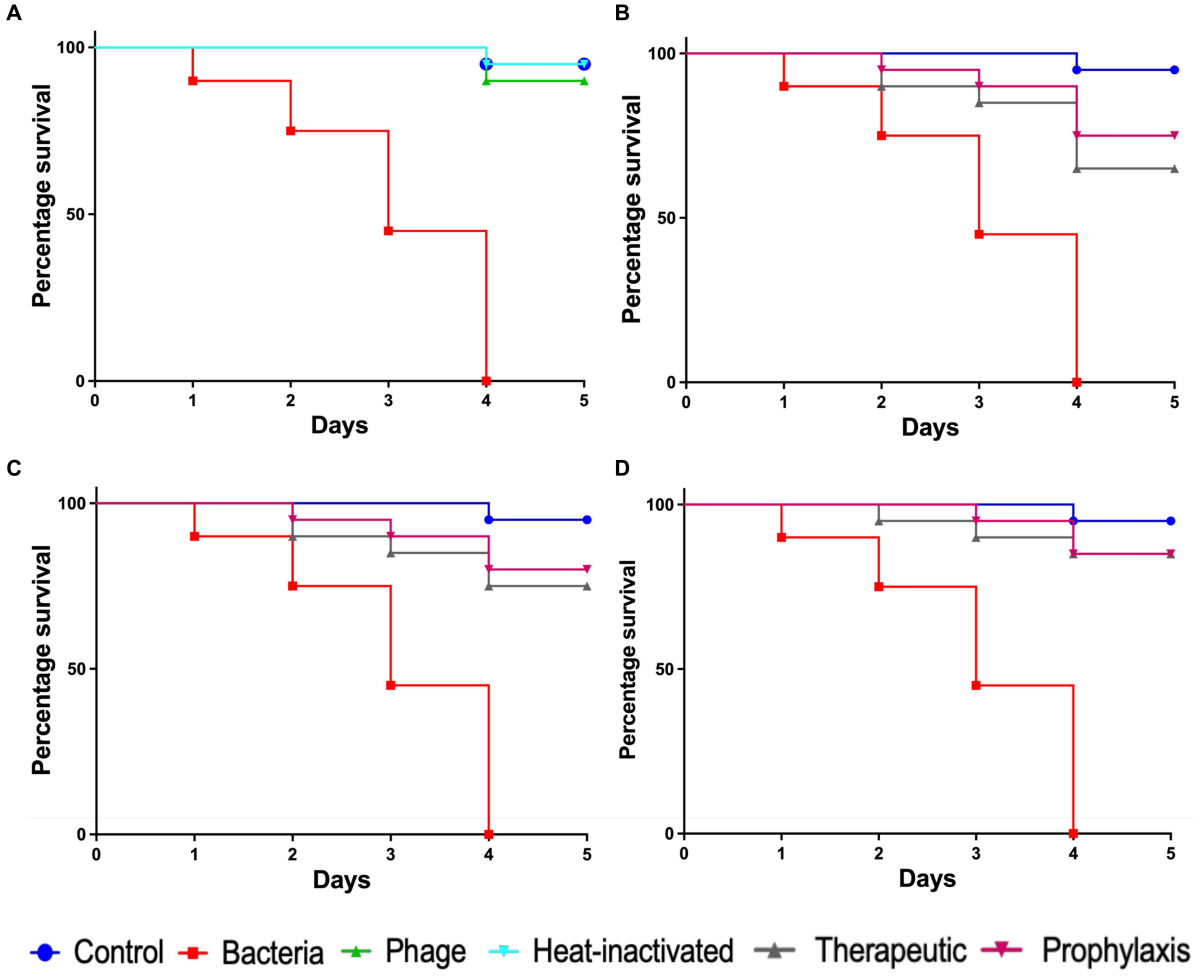
Pathogenicity of *P. aeruginosa* in *C. elegans* and efficacy of *Pseudomonas* phage Motto against pseudomonal infections. The control group consisted of *C. elegans* fed with *E. coli* OP50 and exposed to *P. aeruginosa* (OD_600_ = 0.6) which kills *C. elegans* in a liquid medium. Twenty nematodes were used in each group. Representative survival curves of *C. elegans* following infection by *P. aeruginosa* in **(A)** liquid medium consisting of M9 buffer and *P. aeruginosa* culture, *Pseudomonas* phage, or heat-inactivated bacteria and **(B,C,D)** survival curves of *C. elegans* following infection with *P. aeruginosa* and treatment with phage Motto, therapeutic, and prophylactic treatment. **(B)** Survival curves of *C. elegans* infected and treated with the bacteria–to-phage ratio of 1:1, i.e., 10^5^ CFU/mL and 10^5^ PFU/mL. **(C)** Survival curves of *C. elegans* infected and treated with the bacteria–to-phage ratio of 1:10, i.e., 10^5^ CFU/mL and 10^6^ PFU/mL. **(D)** Survival curves of *C. elegans* infected and treated with the bacteria–to-phage ratio of 1:100, i.e., 10^5^ CFU/mL and 10^7^ PFU/mL. The survival curves were plotted using the Kaplan–Meier method, and the log-rank test was used to analyze the difference in survival rates in GraphPad Prism 9.0. A statistically significant difference (*p < 0.05*) was observed in the treatment groups.

To evaluate the efficacy of *Pseudomonas* phage Motto against pathogenic pseudomonal infections, we employed the recently established *C. elegans* liquid-based assay (Turner et al., 2021). In the infection control group, all the nematodes died within 4 days (Figure 7A). In contrast, heat-inactivated bacteria did not cause any significant effect on the survival of nematodes, with 95% survival observed (Figure 7A). In the group in which the nematodes were exposed to phages but not to bacteria, no decrease in nematode survival was observed. To study the efficacy of the phage treatment, varying multiplicities of infection were tested, i.e., 1:1, 1:10, and 1:100. In the therapeutic treatment group, phage Motto was added after exposure of the nematode to the pathogen for 2 h. In this study, phage Motto was able to increase survival up to 90% within the 5 days observation time when treated at a 1:100 ratio (Figure 7D). The other ratios also increased the survival of nematodes, albeit not as significantly (Figures 7B,C). The more efficient activity was observed from the phage in the prophylactic treatment group, with nematode survival up to 80% after 5 days (Figure 7D). The phage treatment resulted in at least a 500% increase in nematode survival.

## Discussion

In this study, the *Pseudomonas* phage Motto was isolated from Cooum River water in Chennai, Tamil Nadu, India. The genome analysis showed that the phage Motto is closely related to the *Drexlerviridae* family in the order *Caudoviricetes* (Walker et al., 2021). To date, *Pseudomonas* phages with a siphovirus-like morphology have been less well studied. Such phages have been found to have comparably narrow host ranges while also being predominantly lysogenic (Alemayehu et al., 2012; Jalil et al., 2019; Farlow et al., 2020; Grami et al., 2021). However, our study reports a lytic phage with a broad host range among the Drexlerviridae; interestingly, this is the first *Pseudomonas* phage, which is T1-like or Tunalikevirus. T1-like phages are one of the less studied classic T phages, and the previously characterized T1-like phages were mostly infecting *Enterobacteriales* (Cranston et al., 2022). Because T1-like phages can lyse *E. coli* K-12 strains, they are also notorious for contaminating microbiological laboratories (Koonjan et al., 2020). Due to their high adsorption rates, T1-like phages have drawn interest in the phage therapy community (Koonjan et al., 2020).

Motto has a broad host range, is able to infect 64% (*n* = 32/50) of the tested clinical pseudomonal isolates, and is—to the best of our knowledge—one of the least specific phages among the *Drexlerviridae.* However, in most cases, phage resistance is observed *in vivo*, complicating treatment during therapy (Altamirano et al., 2021; Wang et al., 2021). However, phage-resistant mutants have been observed to often display decreased virulence and antibiotic resistance (Altamirano et al., 2021; Wang et al., 2021). Interestingly, we did not observe the emergence of phage-resistant strains under the conditions we tested, making phage Motto a promising candidate for therapy. We also observed rapid absorption and replication, as well as temperature and pH stability, which is considered advantageous for therapy.

We also addressed the question of the safety and efficacy of Motto in tissue culture and in an animal model. We observed no cytotoxic effect of Motto on HEK 293 and RAW 264.7 macrophages when exposed for 24 h. In addition, phage Motto was able to increase the survival of *C. elegans* up to 90% at an MOI of 100 within the 5-day observation time using a recently established liquid-based test platform (Alemayehu et al., 2012). At an MOI of 10, nematode survival was up to 80% (Figure 7). Overall, the phage treatment resulted in at least a 500% increase in nematode survival, illustrating the potential as a candidate for therapeutic applications.

Most lab-based studies of phages that are evaluated or considered for therapy investigate planktonic cells and rarely deal with the issue of biofilms (Latz et al., 2017; Adnan et al., 2020). However, *P. aeruginosa* often causes clinical complications due to the ability to form biofilms, often when present in chronic infections such as cystic fibrosis. Biofilms also can increase antibiotic resistance levels (Stewart and Costerton, 2001; Chegini et al., 2020). Thus, phages that have the ability to disrupt biofilms are of particular interest, to use biofilm-degrading proteins such as depolymerases or the virus as a whole. Motto was able to reduce the biofilm significantly (Figure 4); a similar observation was made with a *Pseudomonas* phage named SL4, a LUZ24-like virus (Latz et al., 2017). Previously characterized T1-like phages were also found to have anti-biofilm activity against *Enterobacteriales* (Oliveira et al., 2017). The same study also reported the impact of exposure time, 3 h and 24 h, in reducing the biofilms. Importantly, our study showed a reduction in strong biofilms after 24 h of treatment, in comparison with previous studies in which no anti-biofilm effect was observed for mucoid clinical strains that produced extensive biofilms (Fong et al., 2017; Latz et al., 2017). Our study does not attempt to obtain absolute quantitative values of biofilm mass but to compare biofilms to each other formed by a single bacterial isolate. As this method allows us to compare whether a sample has more or less biofilm compared to a control in a particular setting under specific conditions, we obtain an indication if the phage indeed reduced the amount of biofilm. For future studies, it might be advantageous to use additional methods to quantify biofilm mass more precisely.

The activity of the phage to disintegrate biofilms is likely caused by enzymatically active proteins on the surface of the virus particle; however, when analyzing the genome of the phage, we were unable to identify obvious candidate genes such as depolymerases or hydrolases (with the exception of the endolysin). Previous studies have shown that tail fiber proteins can exhibit capsule-and/or possibly biofilm-degrading properties (Scholl et al., 2001; Knecht et al., 2020). We identified two tail fiber proteins, ORF21 and ORF28, which could be responsible for the anti-biofilm activity of Motto. Interestingly, ORF28 sequence homologs are tail fibers from *Enterobacteriaceae* phages, with several annotated as endo-N-acetylneuraminidases. Although it is beyond the focus of this study, future experiments could examine these proteins and—for clinical applications—possibly even perform *in vitro* evolution to improve their activity, similar to an approach described here (60).

The previously reported complete DNA sequence of the phage Motto gives only a brief description of the most basic characteristics of the genome (Manohar et al., 2022). Here, we provide details on the 84 ORFs that were predicted, 38 of which have assigned putative functions (Supplementary Table S2). The largest group is comprised of those that form the virus structure and morphogenesis proteins (such as chaperones and tail measure proteins). We identified 11 proteins that putatively interact with, degrade, or modify nucleic acids of either the phage or the host. These proteins include a single-stranded DNA-binding protein (locus_tag = Motto_27) and putative DNA modifying proteins, such as a C-5 cytosine DNA methylase, a deoxynucleotide monophosphate kinase, a phosphoesterase, and a Dam methylase. Lysis-related proteins include a putative holin, a SAR endolysin, and a u-spanin. Interestingly, we also identified a putative super-infection exclusion protein (locus_tag = Motto_24).

The comparative analysis suggests that Motto represents a new species and genus within the family *Drexlerviridae*. To the best of our knowledge, this is the first T1-like phage characterized to infect *Pseudomonas* species.

## Conclusion

We isolated a new phage infecting *P. aeruginosa*, Motto, which exhibits a broad host range, infecting more than 50% of the strains we tested (*n* = 50). In addition to being physically stable and replicating rapidly, two properties make the bacteriophage a promising therapeutic candidate: first, Motto’s activity against biofilms, and second, the absence of phage resistance observed under the conditions we tested. These properties are highly advantageous for the treatment of *P. aeruginosa* infections employing phage therapy.

## Author’s note

As part of this research, data were presented at the Second International Conference on Bacteriophage Research (ICBR), India in 2021.

## Data availability statement

The datasets presented in this study can be found in online repositories. The names of the repository/repositories and accession number(s) can be found in the article/Supplementary material.

## Ethics statement

Ethical approval was not required for the studies on humans in accordance with the local legislation and institutional requirements because only commercially available established cell lines were used. The manuscript presents research on animals that do not require ethical approval for their study.

## Author contributions

PM: Writing – original draft, Validation, Methodology, Formal Analysis, Data curation, Conceptualization. BL: Writing – review & editing, Visualization, Validation, Supervision. DT: Writing – review & editing, Supervision, Software, Methodology. RT: Writing – review & editing, Resources, Project administration, Methodology. MM: Writing – review & editing, Methodology, Formal analysis, Conceptualization. NE: Writing – review & editing, Resources, Methodology, Formal analysis. RN: Writing – review & editing, Validation, Supervision, Resources, Project administration, Investigation. SL: Writing – review & editing, Validation, Supervision, Resources, Project administration, Investigation, Data curation.

## Footnotes

^1^ https://ictv.global/vmr
