# Supplementary material for: In vitro and in vivo evaluation of the biofilm-degrading Pseudomonas phage Motto, as a candidate for phage therapy
Source: Front Microbiol. 2024 Mar 15;15:1344962. doi: 10.3389/fmicb.2024.1344962 (PMC10978715; doi:10.3389/fmicb.2024.1344962)
Supplement: Supplementary file 1 [file Data_Sheet_1.PDF]

Supplementary Material

In vitro **and** ***in vivo*** **evaluation of the biofilm-degrading** ***Pseudomonas*** **phage Motto, as a candidate for phage therapy**

Prasanth Manohar, Belinda Loh, Dann Turner, Ramasamy Tamizhselvi, Marimuthu Mathankumar, Namasivayam Elangovan, Ramesh Nachimuthu*, Sebastian Leptihn*

***Correspondence:** sebastian.leptihn@health-and-medical-university.de OR drpnramesh@gmail.com

Supplementary figures:

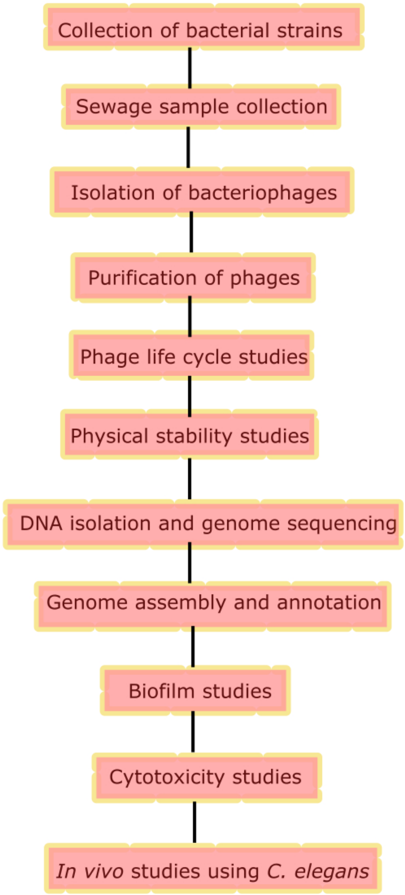

Supplementary figure 1: Methods used in this study is represented as flow chart.

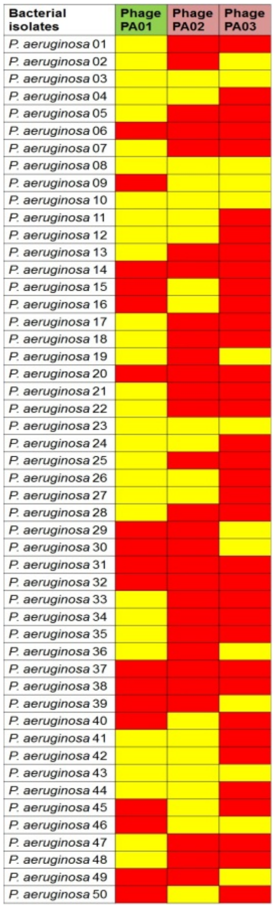

Supplementary figure 2: Host-range infectivity of the isolated phages (n=3) was tested against the pathogenic, mucoid *P. aeruginosa* isolates using spot test and agar-overlay methods. Yellow=active and red=no activity. Phage PA01 (named Motto) (highlighted in green) was characterized in this study.

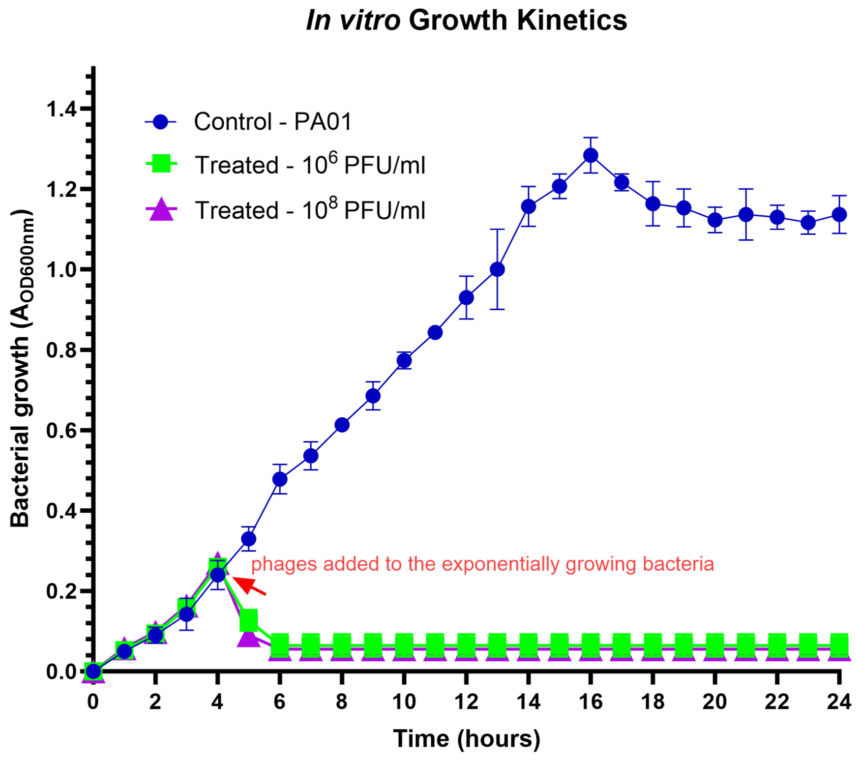

Supplementary figure 3: Growth kinetics of *P. aeruginosa* PA01 with and without phage 'Motto' infection. The difference in the growth pattern of bacteria in the presence and absence of phages was observed based on the average OD value for each timepoint for each group.

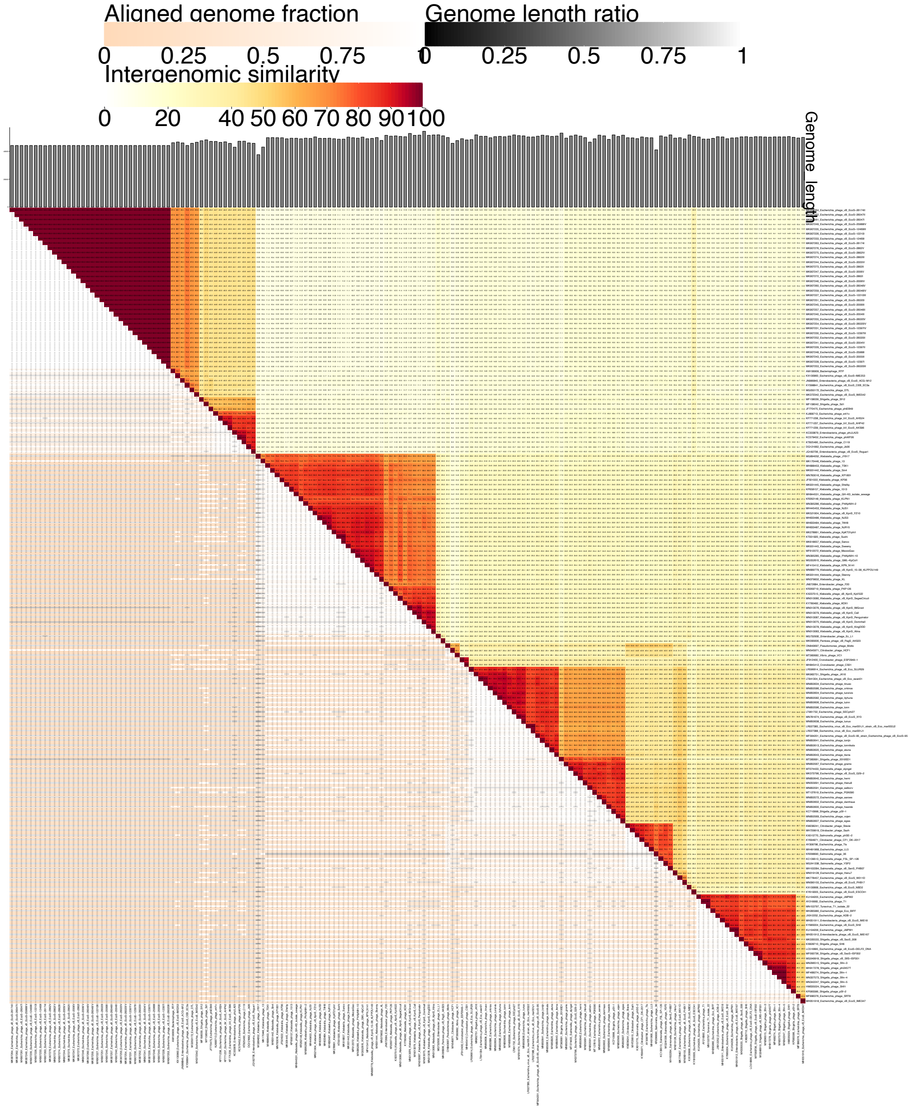

Supplementary figure 4: The relationship between the nucleotide sequences of Motto and other 167 closely-related viral families was classified using VIRIDIC.

Supplementary tables:

Supplementary table 1: Minimal Inhibitory Concentration (MIC) results of the *P. aeruginosa* isolates as obtained from the micro-broth dilution method.

Bacteria	Cefotaxime	Ciprofloxacin	Gentamicin	Meropenem	Tetracycline
P. aeruginosa 01	**64**	2	**16**	**32**	**32**
P. aeruginosa 02	**>128**	**4**	4	0.5	**16**
P. aeruginosa 03	32	**8**	8	2	8
P. aeruginosa 04	8	0.5	**32**	4	4
P. aeruginosa 05	**64**	1	4	2	8
P. aeruginosa 06	8	**32**	**>128**	0.25	**16**
P. aeruginosa 07	4	2	**64**	2	8
P. aeruginosa 08	**>128**	**4**	**16**	**>128**	**16**
P. aeruginosa 09	**>128**	**16**	2	4	4
P. aeruginosa 10	4	**8**	8	8	4
P. aeruginosa 11	2	1	**32**	**16**	**>128**
P. aeruginosa 12	**>128**	0.5	1	4	8
P. aeruginosa 13	16	0.12	4	**64**	**16**
P. aeruginosa 14	32	2	8	8	8
P. aeruginosa 15	**>128**	1	4	**16**	8
P. aeruginosa 16	**64**	**64**	**32**	**32**	**>128**
P. aeruginosa 17	32	1	4	4	2
P. aeruginosa 18	16	2	**64**	8	4
P. aeruginosa 19	**>128**	0.12	1	1	**16**
P. aeruginosa 20	8	0.5	4	0.5	8
P. aeruginosa 21	**64**	2	8	2	4
P. aeruginosa 22	2	2	**16**	8	**32**
P. aeruginosa 23	8	**4**	4	4	**16**
P. aeruginosa 24	**>128**	1	4	**16**	8
P. aeruginosa 25	**64**	**161**	2	8	4
P. aeruginosa 26	16	2	**64**	**32**	0.5
P. aeruginosa 27	**>128**	**8**	**64**	**16**	**32**
P. aeruginosa 28	32	1	8	4	4
P. aeruginosa 29	16	**32**	4	2	8

P. aeruginosa 30	**>128**	1	8	4	2
P. aeruginosa 31	32	1	**16**	**16**	8
P. aeruginosa 32	16	**4**	**32**	**64**	**16**
P. aeruginosa 33	**>128**	2	8	1	4
P. aeruginosa 34	8	2	4	0.25	8
P. aeruginosa 35	16	0.5	**64**	**>128**	**32**
P. aeruginosa 36	16	1	2	2	8
P. aeruginosa 37	4	**32**	**64**	**64**	**16**
P. aeruginosa 38	2	2	1	4	4
P. aeruginosa 39	4	**64**	4	2	2
P. aeruginosa 40	16	1	8	0.5	8
P. aeruginosa 41	32	1	**16**	4	**16**
P. aeruginosa 42	**>128**	**8**	**16**	**>128**	**64**
P. aeruginosa 43	32	**16**	4	4	8
P. aeruginosa 44	**64**	2	4	8	**128**
P. aeruginosa 45	16	0.5	8	**16**	**64**
P. aeruginosa 46	8	2	**16**	4	4
P. aeruginosa 47	**>128**	1	**16**	**16**	**32**
P. aeruginosa 48	8	**4**	2	4	4
P. aeruginosa 49	8	2	4	8	8
P. aeruginosa 50	**64**	2	**16**	8	4

The numerical values in bold represents the resistant isolates and according to CLSI guidelines the resistance breakpoint (µg/mL) of Cefotaxime is ≥64, Ciprofloxacin is ≥4, Gentamicin is ≥16, Meropenem is ≥16, Tetracycline is ≥16. Highlighted in grey are strong biofilm producers.

Supplementary table 2: A list of functional genes identified in the genome of *Pseudomonas* phage Motto. The genes are grouped based on their putative functions.

Morphogenesis and Structure			*DNA-interacting or modifying proteins*			*Host lysis proteins/ others*	
**ORF**	**Putative function**		**ORF**	**Putative function**		**ORF**	**Putative function**
1	large terminase		25	exo-deoxyribonuclease VIII		38	holin
84	small terminase		27	single stranded DNA binding protein		39	SAR endolysin
3	head morphogenesis protein		29	DNA primase/helicase		40	u-spanin
4	major head protein		30	transcriptional regulator			
7	major capsid protein		31	ATP-dependent helicase		24	super-infection exclusion protein
5	capsid decoration protein		32	holliday junction resolvase			
2	portal protein		49	C-5 cytosine DNA methylase			
9	head-to-tail connector complex protein		73	deoxynucleotide monophosphate kinase			
12	minor tail protein		76	GntR-family transcriptional regulator			
13	major tail protein		37	phosphoesterase			
14	tape measure chaperone		33	Dam methylase			
15	tape measure chaperone						
16	tail tape measure protein						
17	minor tail protein						
18	minor tail protein						
19	tail tip protein						

Morphogenesis and Structure			***DNA-interacting or modifying proteins***			***Host lysis proteins/ others***	
20	tail assembly protein						
21	tail fiber protein						
28	phage tail fiber protein						
69	morphogenetic protein						
